# Dr. Goddard on the Arrangement of Sets and Partial Sets of Teeth

**Published:** 1849-04

**Authors:** 


### DR. GODDARD ON THE ARRANGEMENT OF SETS AND PARTIAL SETS OF TEETH.

DIFFERING with some of the profession as to the best mode of constructing sets and parts of sets of artificial teeth, we have (at the solicitation of our professional friends, who have seen us work, and think the method we pursue easier than any they have witnessed, willing also to contribute our mite to the common stock of dental knowledge, if we can in the least degree advance the interest of any, and in order to elicit the views of others) thought best to give a description of our mode, hoping it may induce some who have had more experience to give their opinions upon the subject. We are aware many dentists (eminent and respectable) are wedded to their particular practice, and think it cannot be excelled, and some are even unwilling to test any other, but adhere strictly to their own. We are not, however, among that number, believing some useful hints may be suggested, and knowledge imparted, by the most humble.

What we are about to relate, upon the construction of artificial teeth, is fully understood and practised by many of our profession in this city and other parts of the Union. Some may pursue what they deem a better course, which we hope they will make known for the benefit of all.

It is unnecessary to consume time in speaking of the initiatory steps to be taken in preparing the mouth for artificial teeth, presuming this is, or ought to be, fully understood by every person undertaking this branch of our profession; neither do we purpose speaking of the manner of taking impressions of the mouth, except so far as to give the material we use, the ingredients of which it is composed, and their proportions. This composition, which we think preferable to any now in use, not only for taking impressions of the mouth, but for adapting teeth upon a plate, it being, when slightly heated, very tenacious, adhering firmly to the plate, and retaining the teeth while in the mouth (if care be used to prevent its becoming wet by saliva where the back of the tooth comes in contact with it) sufficiently long for articulating purposes, is made of

| | |
|---|---|
| Best Beeswax, - - - - | 1 lb. |
| Gum Mastic Optimus, - - | 1 ʒ. |
| Whiting, - - - - | ½ ʒ. |

Coloring it red, if wished, with alkanet root, bruised and tied in a piece of cotton cloth. The first two ingredients should be melted slowly, keeping it constantly stirred, adding the whiting last, incorporating it minutely; after being well mixed, remove from the fire; when partly cooled, add any perfume you please, and pour into common plates, when cold, warm the bottom of the plates, and remove the wax; if any sediment, it will be found at the lower side of the wax, which should be scraped off.

When used for impressions, soften the wax in warm water; if required for retaining teeth upon a plate, soften by heat from an alcohol lamp or fire, adapting the teeth as nearly as possible before putting it into the mouth. We have used this composition for the above purposes twelve years; in the meantime, have tried many others which have been highly recommended, but have found none to surpass or equal it.

It is not our intention to trace the work through all its different stages, nor the method of making casts and counter casts—they are various, all leading to the same results; are generally good, and familiar to every dentist; but will suppose the plate swaged to its proper position, and ready to receive the clasps. It may not be amiss here to state, as there is much diversity of opinion as to the manner by which clasps should be made and attached to teeth, by some of our professional brethren, that we invariably prefer and use wide clasps, believing them much less injurious to the teeth they surround, and far more beneficial than any other for the purpose to be accomplished, and cannot agree with the opinions expressed by some writers in the Dental Journal, that "the wide clasp is too unyielding," and apt to destroy the vitality of the tooth by changing its position, thereby paralyzing the periosteum at the points of pressure, and destroying its circulation. We have almost daily instances where narrow (or half-round) clasps are used, of the injury effected by them, the partial or entire excision of the tooth. It is essential in all cases, when artificial teeth are applied, that there should be a perfect adaptation to the parts, the clasps accurately fitted to the teeth, and embrace, if possible, their whole circumference. In some cases, for instance a single tooth, this is not necessary; but we refer to a plate with many teeth upon it, and when attached to the molars.

The manner in which we adapt our clasps insures, in a great degree, a perfect fit; if care be used to trim our metal cast, the plan to correspond with the mouth, they seldom need any alteration; our plan is by forming and swaging them upon the teeth of our metal cast; we then add the plate and stamp it over them; this brings all to an accurate fit; we then change our work to our plaster model, binding the clasps and plate to their proper positions by wire, and solder on our mould. This method we think far superior to that recommended by Dr. Brown, who advises "the clasps to be attached by wax, then lifted from the model, laid upon paper, wax downward, plaster poured over both plate and clasps; when set, dry and solder."

The advantages of the plan we suggest (and presume there are scores of the profession who pursue it) are, the correctness by which the clasps are attached, the facility and accuracy by which it is accomplished. The whole can be soldered in less time than it requires the plaster to set in Dr. Brown's plan; and most certainly, if your model be correct, and your plate and clasps fit, it must also fit the mouth. It is true we destroy the plaster model if this course is pursued, which may be an objection to some—to us it is none; we always take two impressions of the mouth, and two plaster casts. Our plate being partially finished, by filing, &c., we next adapt, as nearly as possible, the teeth before our patient arrives; then, trying the plate in the mouth and adjusting it, remove and adapt the teeth, already selected and fitted, by means of wax or cement, to the plate, arranging the articulation, &c.; remove the whole from the mouth, and place it in a copper saucer, plate downwards, the saucer having been previously half-filled with plaster and sand of consistence of cream; it will imbed itself in the plastering; immediately fill the saucer with plaster and sand, mixed, and let it partially set; before becoming hard, remove the plaster sufficiently to expose the wax, and when perfectly set, remove the wax, and the backs of the teeth are visible. Be particular and not remove entirely the plaster from the tops of the teeth; it is required to retain them in their place, and prevent them from rising when heated, or when foil is forced under them. We are now ready to put on our studs or lining straps, which we do without removing the tooth from the plaster, fit the end of the strap to the plate, and by placing a very thin coating of wax upon its back, press against the pins; the wax will show the indentation made by the pins, and the places and positions where to punch the holes; this being done, countersink the holes slightly, file your back as you wish, replace upon the tooth, place your saucer upon a leaden slate on its side, and lightly rivet, cutting off, however, any superfluous pin before riveting. Your teeth are firmly and securely imbedded in a hard substance, equally surrounded upon all sides, and are in no danger of breaking by slightly riveting. After having completed lining your teeth, file the heads of the pins down smoothly, which can be as easily done as if they were already soldered, they are so securely fixed. This plan is much better than the one recommended by Dr. Brown, of removing the tooth and backing it: we never remove a tooth; and if, by accident, one becomes loose, it is a source of trouble to us. Borax your work well; place on your solder, filling up any interstices which may happen with gold foil, and place in your oven. We endeavor to complete all plate-work thus far by night, letting it remain in the oven until morning, at which time it is thoroughly dry; place then upon a charcoal fire in our furnace; in half an hour, or less time, if in haste, the work is heated to redness; removing it then into a copper holder, fitted for receiving the saucer; place under the blow-pipe, and in half a minute all is soldered in a perfect manner; cover immediately the saucer with a larger one; if block work, fill the saucer with pulverized charcoal first, then cover with a large one and let it cool. We seldom crack a tooth, or block work, and the whole resembles a casting.*

These remarks have been hastily penned, not so much to instruct, (for they may be generally understood by all,) as for the purpose of inducing suggestions of any better method of accomplishing the desired end. Many of the profession, I am aware, pursue a different plan, and several I know have tried other methods, who now give this a decided preference, and think it far superior to any other, in point of safety to the work, economy in time, and facility to the dentist.—Lou. Ed.

* We would remark, the furnace and blow-pipe in use is one constructed by our friend, Dr. R. Somerby, of Louisville, Ky., and is one of the most complete instruments for dental purposes we have ever seen, and should be in the laboratory of every scientific dentist. A full description of it can be seen in the second number of the fifth volume of the Dental Journal.

